# Treatment of atopic dermatitis using non-thermal atmospheric plasma in an animal model

**DOI:** 10.1038/s41598-021-95471-z

**Published:** 2021-08-09

**Authors:** Ik Jun Moon, Mi Ra Yun, Hae Kyeong Yoon, Keon Hee Lee, Sun Young Choi, Woo Jin Lee, Sung Eun Chang, Chong Hyun Won

**Affiliations:** 1 Department of Dermatology, Asan Medical Center, University of Ulsan College of Medicine, Seoul, Republic of Korea; 2 Department of Medical Science, Asan Medical Institute of Convergence Science and Technology, Asan Medical Center, University of Ulsan College of Medicine, Seoul, Republic of Korea; 3 Asan Institute for Life Sciences, Asan Medical Center, Seoul, Republic of Korea; 4 Department of Dermatology, Ilsan Paik Hospital, Inje University College of Medicine, Gyeonggi-do, Republic of Korea

**Keywords:** Cell biology, Medical research

## Abstract

Cold atmospheric plasma (CAP) has been incorporated into various fields, including promotion of cutaneous wound healing. Atopic dermatitis (AD) is a chronic cutaneous condition characterized by inflammation-induced skin wounds and impaired skin barrier function. To investigate whether CAP may improve AD using an animal model. *Dermatophagoides farinae* extracts (DFE)-induced murine models of AD were used in this study. The plasma-treated group received a total of 6 CAP treatments during 2 weeks, while the control group did not receive any treatment. Differences in dermatitis severity, transepidermal water loss (TEWL), serum level of immunoglobulin (Ig) E and epidermal thickness were evaluated in both groups. The dermatitis severity was significantly improved by CAP treatment. TEWL was lower in the plasma-treated group compared with the non-treated control group. Serum Ig E dropped significantly after treatment with CAP. Difference in epidermal thickness of the ear skin was not significant between the plasma-treated and non-treated groups. Localized treatment of AD with CAP decreases dermatitis severity, TEWL, and serum Ig E level. These results show CAP’s potentials as a novel therapeutic modality for AD.

## Introduction

Atopic dermatitis (AD) is a chronic inflammatory cutaneous condition that not only causes severely distressing symptoms such as itching and burning, but also results in scarring and pigment alteration of the skin. Patients with AD have disrupted skin barrier; the essential water content of the skin evaporate while the skin becomes highly susceptible to both physical and chemical injuries^1^. A good number of treatment options for AD are available, including topical corticosteroid, topical calcineurin inhibitors, and systemic agents such as corticosteroids, cyclosporine, and azathioprine. A combination approach is usually taken in the treatment of AD, and combined topical and systemic treatments tend to show good control of disease. However, the possibility of adverse events and toxicity related to therapeutic agents is always a concern. Because AD tends to have a very long disease duration, active research focused on the development of novel treatment modality which is effective and safe, with minimal risk of systemic toxicity, is ongoing. Furthermore, AD tends to commonly involve certain body regions, such as antecubital and popliteal areas, and relapse is frequent in these areas. Therefore, there is a particularly strong need for effective localized treatment.

Atmospheric pressure non-thermal or ‘cold’ plasma is a relatively recent technology that serves medical purposes as a disinfecting and bleaching technique^2–4^. The clinical application of cold atmospheric plasma (CAP) has been reported in a number of studies, but the value of CAP treatment for AD has not been investigated thoroughly. A previously conducted case study reported clinical improvement of itch caused by AD after CAP treatment^5^. In contrast, no significant reduction of itch after CAP treatment was observed in a randomized placebo-controlled study^6^. Therefore, there is still controversy on the therapeutic effects of CAP for the treatment of AD. Recently, there have been reports on CAP’s ability to promote cutaneous wound healing after physical injury^7^. The evidence that it stimulates keratinocyte and fibroblast migration, alters cytoskeletal dynamics in vivo, and accelerates healing in a murine model of skin wounds led to the speculation that CAP may also improve skin barrier function as well as the overall disease severity of AD. In this study, we examined the effect of CAP treatment on the clinical severity, molecular profile, and skin histology in a murine model of AD.

## Materials and methods

### Murine model of atopic dermatitis (AD)

**Figure 1:**
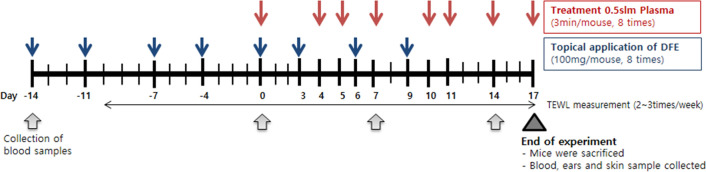
Experimental design for the in vivo study using the mouse model of atopic dermatitis.

The protocol is summarized in Fig. 1. Twenty-eight male NC/Nga mice (Central Lab Inc., Seoul, Korea), 5 weeks after birth, were used in the study. Mice were divided into three groups: normal control (n = 4), AD (n = 12), and AD + plasma (n = 12). The back and the skin around the ears were shaved using a depilatory cream. During the experiment, no intervention was made on the normal control group. For both AD and AD + plasma groups, 100 mg of *Dermatophagoides Farinae* Extracts (DFE) (BIOSTIR Inc., Osaka, Japan) were applied twice weekly for two weeks (from Day –14 to Day –4). Starting from Day 0, DFE was applied every three days until Day 9.Before every application, the hair of mice was removed with a depilatory cream. All mice were reared under specific pathogen-free conditions. All animal experiments were carried out in compliance with the ARRIVE guidelines (https://arriveguidelines.org), the Institute of Laboratory Animal Resources (ILAR) guidelines, and the Principles of Laboratory Animal Care formulated by the Institutional Animal Care and Use Committee (IACUC) of the Asan Institute for Life Sciences, Asan Medical Center, Seoul, Korea. All experimental protocols were approved by the IACUC of the Asan Institute for Life Sciences.

### Physiochemical characterization of the cold atmospheric plasma

**Figure 2:**
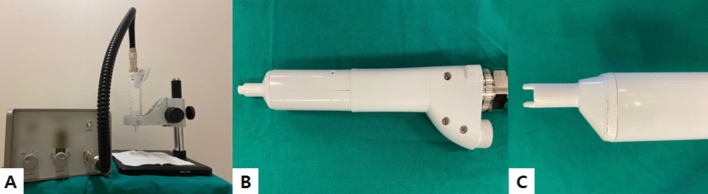
Photographs of the investigated cold atmospheric plasma device. (**A**) During the treatment, the handpiece was held static using a clamp. (**B**) The handpiece is designed similar to other conventional energy-based devices and can be easily held manually. (**C**) The tip of the handpiece is equipped with a 5 mm-long plastic cover, which keeps the distance from the plasma jet and the treated area constant.

The CAP device employed in this study was developed and built by MediPL, Inc, Seoul, Korea (model name: RADIX-0502). Photographs of the device are presented in Fig. 2. The device uses argon gas and the handpiece emits a single jet of plasma. The power of plasma jet used was 1.5 W. The flow of argon gas could be controlled from 0 to 5 l/min. Throughout the experiment, the argon gas flow was kept at 0.5 l/min.

### CAP treatment

For the AD + plasma group, plasma treatment was delivered seven time in total, starting from Day 0 and until Day 17. CAP treatment was performed on Day 0, 4, 5, 7, 10, 11, 14, and 17. Mice in the AD + plasma group were placed under gas anesthesia using isoflurane, and the handpiece of the device was placed static on their dorsal skin using a clamp so that the tip of the handpiece could make contact with the dorsal skin. Two different areas (the occiput and the apex of the back) were treated for 3 min each.

### Assessment of dermatitis severity

The severity of AD was assessed using a Dermatitis Severity Score (DSS), which consists of degree of erythema/hemorrhage, edema, scale/dryness, and excoriation/erosion. For each category, a score of 0 (none), 1 (mild), 2 (moderate), and 3 (severe) was given by the observer. The sum of all four categories represented the overall dermatitis severity. For each mouse, DSS was assessed by three independent investigators. The average total DSS scores were used for analysis.

### Measurement of transepidermal water loss

Transepidermal water loss (TEWL) was measured using GPSkin Barrier device (GPOWER Inc., Seoul, Korea) on the dermatitis-induced skin of mice after shaving and under anesthesia. If CAP treatment was to be performed on the same day, TEWL measurement was done before the treatment to minimize the drying effect of CAP. Measurements was repeated three times, and the average TEWL score was used for analysis. Measurements were performed on day 0, 7, and 14.

### Measurement of serum immunoglobulin E

Blood samples from the retro-orbital sinus of mice were collected before and two weeks after plasma treatment using heparinized capillary tubes. Serum samples were obtained by centrifugation at 15,000 rpm for 20 min and were kept refrigerated at − 80 °C. The levels of immunoglobulin (Ig) E were measured at room temperature using Mouse Ig E ELISA kit (Shibayagi Inc., Shibukawa, Japan) according to the manufacturer’s instructions.

### Measurement of epidermal thickness

The skin samples were fixed in 10% buffered neutral formalin and embedded in paraffin. These specimens were cut into 6 μm sections, and serial sections were stained with hematoxylin & eosin (H&E) to measure the epidermal thickness. Epidermal thickness was defined by the distance from the peak of the stratum corneum to the dermoepidermal junction. The epidermal thickness was measured at five random spots, and the mean thickness was calculated.

### Statistical analysis

For analyzing DSS scores, TEWL values, and serum Ig E levels, one-way analysis of variance (ANOVA) and post hoc analysis (Tukey’s test) were performed. For comparison of the epidermal thickness between groups, Student’s T-test was done. All statistical analyses were performed using a statistical software package (SPSS, version 22.0; SPSS Inc., Chicago, IL). Differences were considered statistically significant when p < 0.05.

## Results

### Change in dermatitis severity after CAP treatment

**Figure 3:**
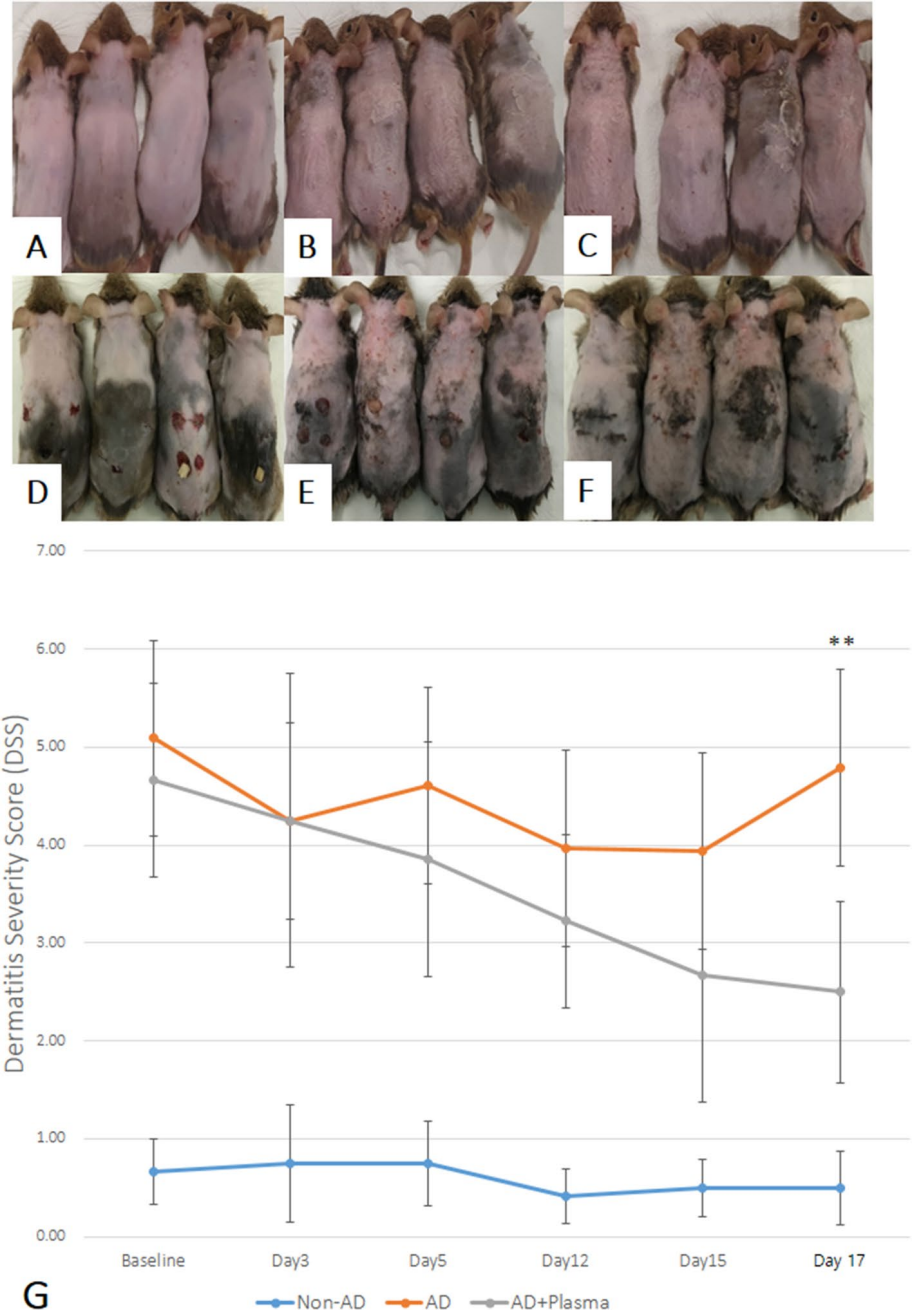
The effect of atmospheric pressure cold plasma treatment on the Dermatitis Severity Score (DSS) of atopic dermatitis-induced mice. Photographs of the dorsal skin of mice in the (**A**,**D**) non-sensitized control group (Non-AD), (**B**,**E**) AD group, where induction of atopic dermatitis was done without plasma treatment, and (**C**,**F**) AD + Plasma group, where atopic dermatitis was treated with cold plasma (**A**–**C**: Day 0; **D**–**F**: Day 17). (**G**) The mean total DSS scores of both the AD group and the AD + plasma group serially decreased after induction of AD-like lesions. Their mean DSS score differed significantly on Day 17, where the mean DSS score of the AD + Plasma group was lower than that of the AD group. **P < 0.001.

The severity of dermatitis was assessed using the predefined dermatitis severity score (DSS) on day 0 (baseline), 3, 5, 12, 15, and 17. After the induction of atopic dermatitis (AD)-like skin lesions, both the non-treated AD group and the plasma-treated AD group (AD + plasma) demonstrated notably elevated DSS scores compared with the non-sensitized control (Fig. 3A–C). Compared to the AD group, where no intervention was made after induction of eczematous skin lesions, the AD + plasma group demonstrated more rapid decrease in DSS through day 17. (Fig. 3D–F) The difference in the mean DSS was statistically significant on Day 17 (Fig. 3G, p < 0.001).

### Change in transepidermal water loss (TEWL) after CAP treatment

**Figure 4:**
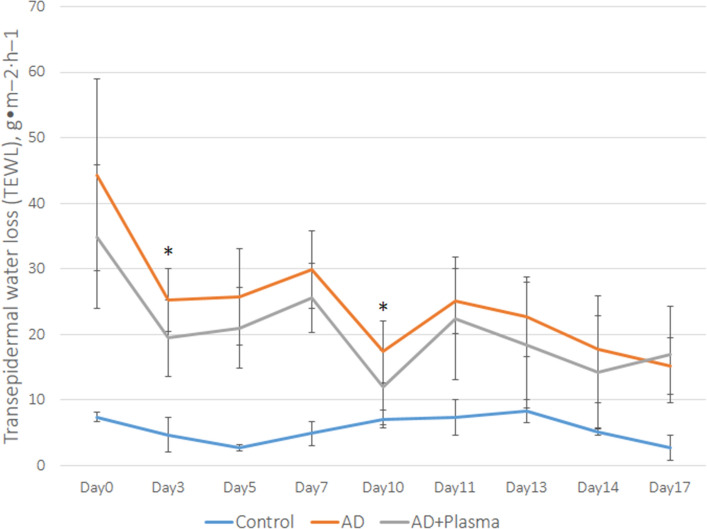
The effect of plasma treatment on the transepidermal water loss (TEWL) of atopic dermatitis-induced mice. While an overall fluctuation of TEWL scores was observed in all three groups, TEWL of the AD + plasma group was generally kept lower than the non-treated AD group. Statistically significant difference in TEWL between AD and AD + plasma groups was observed on Day 3, and Day 10. *P < 0.05.

Measurement of transepidermal water loss (TEWL) was performed at twelve different time points during the experiment. On Day 0, both plasma-treated and non-treated groups showed significantly higher TEWL values compared to the non-sensitized control (Fig. 4). Although TEWL of the AD group was higher than the AD + plasma group on Day 0, the difference was not statistically significant. In the course of the experiment, TEWL of the AD + plasma group was kept lower than that of the AD group. The differences in their TEWL values were found to be statistically significant at two time points: on Day 3 and Day 10 (p = 0.022 and p = 0.027, respectively). Even though the TEWL values of AD + plasma group were constantly lower than the AD group, they were still significantly higher when compared with the non-sensitized control group.

### Change in serum levels of Ig E after CAP treatment

**Figure 5:**
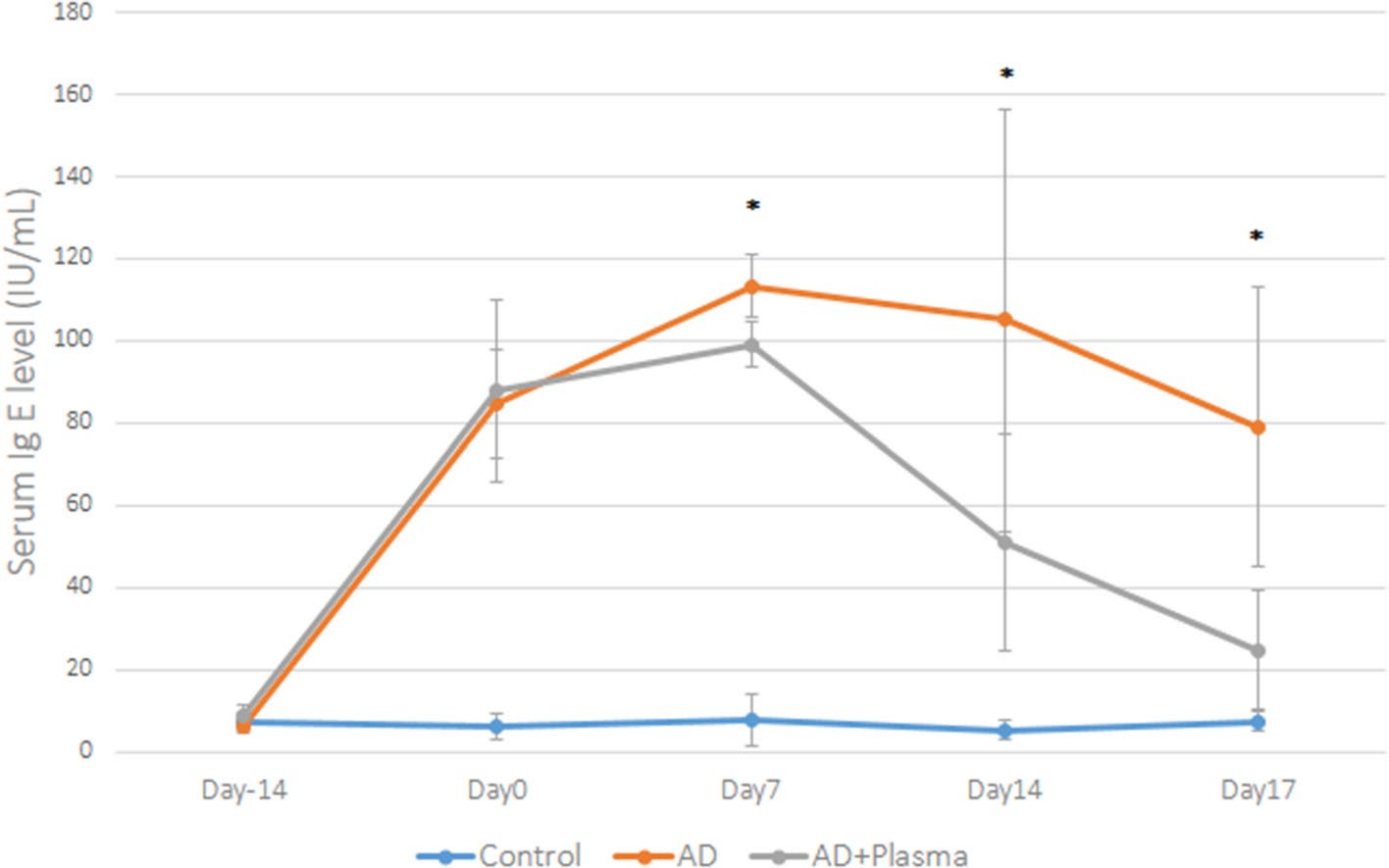
Changes in serum Ig E levels with or without plasma treatment. After induction of atopic dermatitis-like skin lesions in mice, serum Ig E levels were notably elevated (Day 0). Compared to the non-treated AD group, the AD + plasma showed significantly lower levels of serum Ig E starting on Day 7, lasting to the end of the experiment on Day 17. *P < 0.05.

On day 0, both the AD group and AD + plasma group showed comparable levels of serum Ig E levels, which were significantly elevated compared to the control group (p < 0.001 for both groups) (Fig. 5). On Day 7, serum Ig E levels of AD + plasma group was significantly lower than that of AD group (p < 0.001). Statistically significant differences in serum Ig E levels were also observed on both Day 14 and Day 17 (p = 0.01 and p < 0.001, respectively). Despite notably lowered serum Ig E values of the AD + plasma group on Day 17, they were still significantly elevated compared to those of the control group (p = 0.002).

### Effect of CAP treatment on epidermal thickness of ear skin

**Figure 6:**
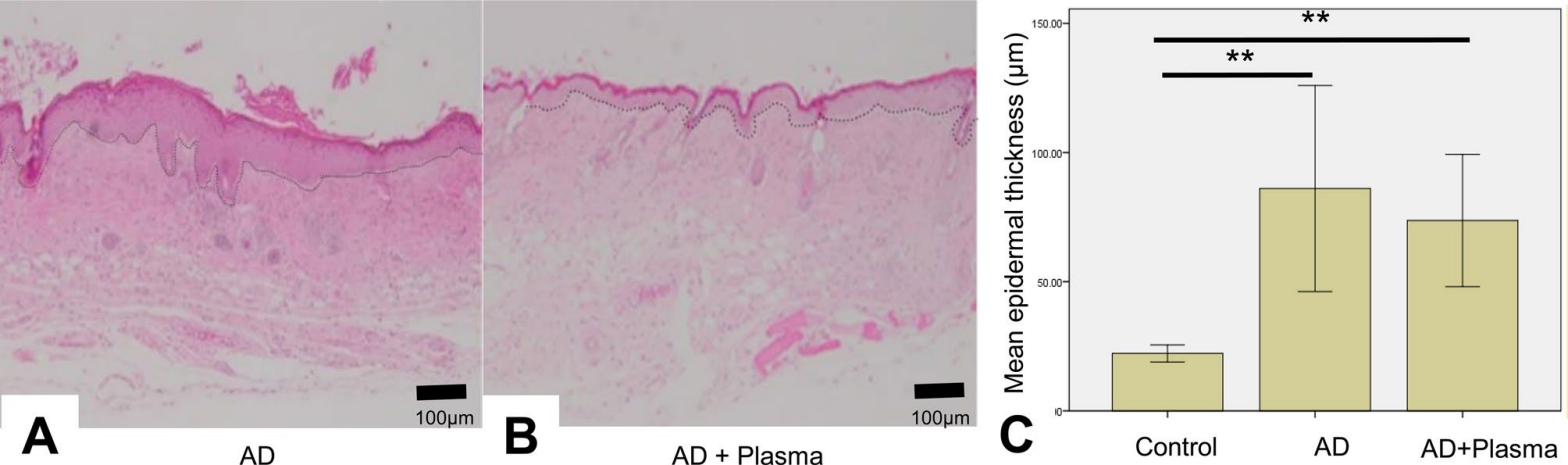
The effect of plasma treatment on the epidermal thickness of atopic dermatitis-induced mice. On Day 17, the ear skin of the mice were sampled and tissue sections with hematoxylin and eosin (H&E) stains were examined for the thickness of the epidermis. The mean epidermal thickness of both (**A**) the AD group and (**B**) the AD + plasma group were significantly thicker than the control group where induction of AD-like dermatitis was not performed. (**C**) Although the epidermal thickness of plasma-treated group was thinner than the non-treated group, the difference was not statistically significant. (H&E, original magnification × 40) **P < 0.001.

Considering that AD as well as other eczematous skin conditions result in thickened epidermis, the epidermal thickness was measured in order to visualize the therapeutic effect of CAP treatment. On day 17, the epidermal thickness of the ear skin of the mice were measured after making hematoxylin & eosin (H&E) stained slides using biopsy specimens. For each slide, the dermoepidermal junction was first manually delineated by a dotted line as shown in Fig. 6A,B. For both AD and AD + plasma groups, the epidermis of the ear skin was found to be significantly thicker than that of the control group (Fig. 6C, p < 0.001 for both groups). The mean epidermal thickness of the AD + plasma group was lower compared to the AD group (72.28 μm vs 88.7 μm), but this difference was not statistically significant (p = 0.39).

## Discussion

In this study, the effect of cold atmospheric plasma (CAP) treatment on a murine model of atopic dermatitis (AD) was investigated. The dermatitis severity was significantly improved by CAP treatment. Transepidermal water loss (TEWL) was lower in the CAP-treated group compared with the non-treated group. Serum level of immunoglobulin (Ig) E dropped significantly after treatment with CAP. Finally, the epidermal thickness of the CAP-treated group was thinner than the non-treated group, but this difference was not statistically significant.

Although a wide array of treatment options is available for AD, each of them carries a potential for side effects. Even topical medications possess risk of both local and systemic side effects in response to absorption of active ingredients. Thus, patients seek therapeutic approaches that does not involve systemic exposure to potentially noxious compounds. The application of CAP on the skin is relatively new. Its efficacy has been tested mainly on wounds, where it proved to accelerate healing without remarkable adverse events^8,9^. Nowadays CAP treatment is becoming a promising treatment modality for various conditions, including different types of cancer^10,11^. The basic mechanism of CAP consists of generation of reactive species. Both oxygen-and nitrogen-based radicals are produced, which lead to an intricate chemical process to finally result in a change in cellular and tissue biology^12,13^.

The results of this study indicate that CAP treatment could effectively reduce the severity of AD. Among other findings, notable decrease in the dermatitis severity clearly demonstrate the effect of CAP treatment. Though statistically significant improvement was observed on the final day of assessment, this can be interpreted as a rapid drop in dermatitis severity considering the chronic course of AD. In fact, most animal studies on topical as well as systemic agents for treatment of AD have observed significant improvement starting from day 15 at the earliest, with some studies reporting emergence of significant change in dermatitis severity by week 6^14–16^. Therefore, our results suggest that CAP treatment could be a fast-acting treatment option for AD. We also believe that longer duration of experiment with additional sessions of CAP treatment would have demonstrated sustained, if not more pronounced, therapeutic effect of AD in terms of DSS.

We have also evaluated the effect of CAP on the TEWL of AD-induced mice. TEWL is prone to substantial variation depending on multiple factors such as the hydration status of the measured subject, it is closely related to the skin barrier function. As shown in Fig. 3, TEWL values of the AD + plasma group was significantly lower than that of the AD group at different time points. We believe that the effect of CAP treatment on TEWL is closely linked to its ability to promote wound healing^7,8,17,18^. Because TEWL reflects the epidermal integrity, epithelial regeneration by proliferation of both epidermal keratinocytes as well as dermal fibroblasts would restore the function of the epidermis as a physical barrier and consequently reduce water loss. In terms of disrupted skin barrier function in AD, decreased expression of epidermal tight junction proteins has been associated with AD^19^. Low expression levels of tight junction proteins result in impaired skin barrier function. Defective skin barrier not only increases TEWL but also makes the skin susceptible to allergenic, noxious external substances. We did not assess the expression of tight junction proteins in the present study. However, we suggest that assessment of tight junction protein expression would further support the potential of CAP treatment in restoring the impaired skin barrier in AD.

We also looked at the effect of CAP on the epidermal thickness of ear skin as an indirect measure of pruritus severity because repetitive scratching results in thickened epidermis, known as lichenification. Reported in the vast majority of patients with AD, pruritus is the major debilitating symptom, often difficult to alleviate. Despite our expectation that CAP treatment could result in decreased epidermal thickness, the difference between the CAP-treated group and the non-treated group was not significant. Several authors have reported reduction of lesional epidermal thickness by novel therapeutic options for AD^20–22^. However, notable decrease in epidermal thickness was observed by no earlier than week 4 after treatment initiation. This may be due to the fact that both appearance and resolution of lichenification is a slow process, requiring several weeks before one can observe significant change in the epidermal thickness. Hence, we suspect that the duration of our experiment was probably too short to observe definite changes in the epidermal thickness. Moreover, although we did not observe a significant change in epidermal thickness following CAP treatment, reduction in DSS shows its potentials to reduce epidermal thickness because dermatitis severity and intensity of pruritus show a positive correlation^23^.

The mechanism by which CAP shows a therapeutic effect on AD is still unclear. We hereby suggest two possible modes of action for the treatment of AD using CAP: promotion of cutaneous wound healing and reduction of microbial burden. As mentioned earlier, there exist numerous reports on accelerated cutaneous wound healing by CAP treatment. Faster recovery of cutaneous wounds, mostly resulting from scratching, would have led to both reduced dermatitis severity and recovery of skin barrier function demonstrated by decreased TEWL. Moreover, improvement of skin barrier function can be coupled with the finding that the serum Ig E level dropped significantly in the CAP-treated group. It is possible that the CAP-treated mice had lower serum Ig E levels because healthy skin barrier makes the epidermis less permeable to potential allergens and reduces the risk of systemic allergic reaction. Another possible mechanism by which CAP treatment shows therapeutic effect on AD is by altering the skin surface microbiome. Microbiome dysbiosis in the skin has been found to be closely related to AD^24^. In view of the classical antiseptic property of plasma treatment, CAP could restore the imbalance in skin microbiota related to AD by reducing noxious microbial burden. If so, CAP treatment would possess a notable advantage over other antimicrobial treatments because it does not seem to have risk of antibiotic resistance and does not irritate the skin as opposed to antiseptic agents such as alcohol. Nonetheless, whether skin microbiome was altered by CAP treatment and whether CAP can selectively eliminate microbes such as *Staphylococcus aureus* and *Malassezia spp*., which are well-known to be related to AD exacerbation, have not been investigated in this study^25^. Therefore, analysis on the alteration of skin microbiome following exposure to CAP seems necessary to confirm our speculations.

To date, there exist on a few studies on the application of CAP for the treatment of inflammatory skin diseases. Although we have observed promising results regarding the beneficial effects of CAP treatment on AD, whether it has therapeutic potentials for other inflammatory skin conditions is questionable. In fact, conflicting results have been reported for the treatment of psoriasis using CAP^26–28^. We believe that AD and psoriasis are quite different in terms of clinical, histological, and molecular characteristics. For instance, psoriasis is characterized by aberrant keratinization with epidermal hyperplasia, but key features of AD such as disrupted skin barrier with increased susceptibility to microbial insults are rarely observed in psoriasis. For this reason, restoration of skin barrier, which is believed to be the main mechanism involved in the treatment of AD using CAP, may not have a therapeutic effect on psoriasis. Furthermore, the penetration depth of CAP into the skin could be a major factor that causes the difference in response to CAP exposure between the two dermatologic conditions. To the best of our knowledge, how deep CAP can penetrate either intact or disordered skin has not yet been investigated. However, CAP is thought to be able to penetrate the skin, considering the fact that human skin is permeable to small molecules. In psoriasis, the causative inflammatory processes not only take place in the epidermis but also in the dermis, whereas the key inflammatory changes in AD take place mainly in the epidermis. Moreover, CAP is likely to penetrate deeper in AD-affected skin than psoriatic skin because epidermal barrier is markedly disrupted in AD. As a result, epidermal exposure to CAP may positively affect the skin inflicted with AD but not psoriasis. In addition, it should be noted that studies using CAP not only reported conflicting results but also differed in the type of gas used to generate CAP. Although CAP can be generated using different neutral gases including argon, nitrogen, and helium, the differences in the biologic effects depending on the gas source remain largely unknown. Hence, future studies comparing the effects of CAP formed using different gas sources are warranted in order to determine the best choice of gas source for a particular condition.

Despite promising findings suggesting the potential of CAP as a therapeutic option for AD, the actual application of CAP treatment in real life seems to have a number of obstacles. First, AD is almost invariably a generalized skin condition rather than a localized disease. Thus, patients tend to have multiple, widespread lesions. Unfortunately, treatment with CAP involves several minutes of exposure. This may make CAP treatment a wearisome procedure if the patient has widespread disease. Second, unlike other energy-based dermatologic treatments, it is difficult to quantify the effective amount of CAP. Consequently, defining the best mode of treatment for the patient becomes a difficult task. CAP treatment has been reported to have a bimodal physiological effect^29,30^. That is, the effect of CAP treatment is not proportional to its effective dose. Beneficial effects of the CAP treatment is observed up to a certain point, but beyond this point, detrimental effects are observed instead. Hence, objective quantification of CAP is truly critical. So far, most studies described the mode of CAP treatment using multiple variables, such as the power voltage and the flow of gas. However, these variables may not be sufficient to characterize the mode of CAP delivery in detail, because other factors including the surface area exposed to CAP, distance from the power source may differ depending on the device used. Thus, we expect that a standard description of CAP treatment be set in the near future to facilitate communication among investigators and objective interpretation of the data. Lastly, the nature of investigations using animal models limits the clinical interpretation of our results. Therefore, a clinical study involving human participants is necessary to confirm the clinical benefits of CAP treatment for AD.

In conclusion, this animal study suggests the potentials of CAP as a novel therapy for AD, as demonstrated by improvements in the severity of dermatitis, TEWL, and serum Ig E level induced by CAP treatment.
